# Glutamate, Gangliosides, and the Synapse: Electrostatics at Work in the Brain

**DOI:** 10.3390/ijms25168583

**Published:** 2024-08-06

**Authors:** Henri Chahinian, Nouara Yahi, Jacques Fantini

**Affiliations:** Faculty of Sciences, Department of Biology, University of Aix-Marseille, INSERM UA16, 13015 Marseille, France; henrichahinian@gmail.com (H.C.); nouara.yahi@univ-amu.fr (N.Y.)

**Keywords:** sphingolipid, ganglioside, lipid raft, neuron, astrocyte, brain, glutamate, synapse, electrostatic surface potential, receptor

## Abstract

The synapse is a piece of information transfer machinery replacing the electrical conduction of nerve impulses at the end of the neuron. Like many biological mechanisms, its functioning is heavily affected by time constraints. The solution selected by evolution is based on chemical communication that, in theory, cannot compete with the speed of nerve conduction. Nevertheless, biochemical and biophysical compensation mechanisms mitigate this intrinsic weakness: (i) through the high concentrations of neurotransmitters inside the synaptic vesicles; (ii) through the concentration of neurotransmitter receptors in lipid rafts, which are signaling platforms; indeed, the presence of raft lipids, such as gangliosides and cholesterol, allows a fine tuning of synaptic receptors by these lipids; (iii) through the negative electrical charges of the gangliosides, which generate an attractive (for cationic neurotransmitters, such as serotonin) or repulsive (for anionic neurotransmitters, such as glutamate) electric field. This electric field controls the flow of glutamate in the tripartite synapse involving pre- and post-synaptic neurons and the astrocyte. Changes in the expression of brain gangliosides can disrupt the functioning of the glutamatergic synapse, causing fatal diseases, such as Rett syndrome. In this review, we propose an in-depth analysis of the role of gangliosides in the glutamatergic synapse, highlighting the primordial and generally overlooked role played by the electric field of synaptic gangliosides.

## 1. Introduction

In the central nervous system, information is propagated by two distinct mechanisms: the nerve impulse and the synapse. The nerve impulse and electrical conduction travel along the axon to the end of the neuron. At this level, the continuity of the electrical signal suddenly cuts off, which requires a suitable mechanism to allow the transfer of information between two neurons. To solve this problem, nature has put in place a specialized structure, the synapse [1,2]. The functioning of the synapse is based on the transfer of information between two neurons, the transmitter (pre-synaptic neuron) and the receiver (post-synaptic neuron). In this case, electrical conduction is replaced by chemical information transferred by neurotransmitters. The basic principle of synaptic transmission involves the travel of neurotransmitters in the synaptic space, bathed in water and ions [3]. Physicochemical criteria control the functioning of the synapse by acting directly on neurotransmitters. Fundamental biological parameters then come into action: water, time, and the electrostatic surface potential of biomolecules [4]. In this context, the post-synaptic membrane plays an essential role in the bioavailability and fate of neurotransmitters.

## 2. Electrostatic Surface Potential of Neurotransmitters

**Figure 1:**
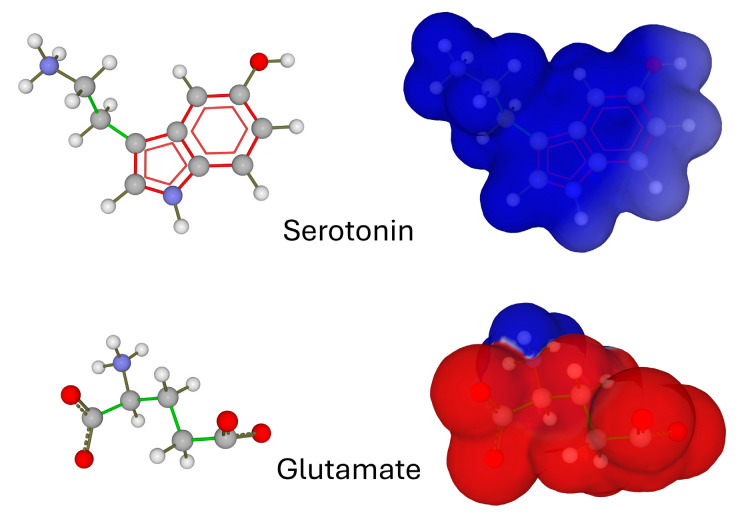
Chemical structure (**left** panels) and electrostatic surface potential (**right** panels) of serotonin and glutamate. Electronegative surfaces are in red, electropositive in blue (the chemical structure of the neurotransmitters is made visible by transparency).

The electrostatic surface potential is a largely underestimated parameter of neurotransmitter activity [5,6]. To illustrate this point, we cannot find a better example than comparing this property for serotonin and glutamate (Figure 1). These two neurotransmitters have opposite electrical charges at a physiological pH, due to the presence of ionizable groups: the amine group for serotonin [7], the amine group and two carboxylate groups for glutamate [8]. The net electrical charge of these neurotransmitters in the synaptic cleft is, therefore, positive for serotonin and negative for glutamate. This greatly affects the electrostatic surface potential of the two molecules. Thus, serotonin has globally positive potential, while that of glutamate is highly negative (Figure 1).

## 3. Electrostatic Surface Potential of Lipid Rafts

**Figure 2:**
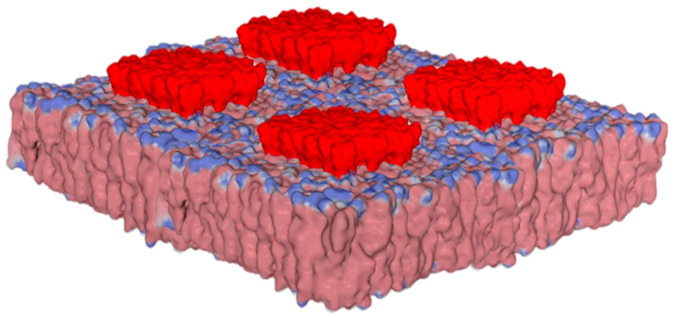
Biochemical organization and surface potential of lipid rafts. Note that raft microdomains protrude at the membrane surface, with a strong electronegative field due to anionic gangliosides (colored in red). The surrounding lipids are a mixture of phosphatidylcholine and cholesterol. Modified from ref. [15] (published by MDPI under the terms and conditions of the Creative Commons Attribution (CC BY) license).

Lipid rafts are specialized plasma membrane microdomains enriched in cholesterol and sphingolipids [9]. Among sphingolipids, gangliosides containing anionic sialic acids are especially abundant in the central nervous system [10,11]. The enrichment of gangliosides gives lipid rafts a high density of negative electrical charges, which makes these areas of the membrane particularly suitable for selectively orienting ligands, such that cationic areas are directed toward the membrane, while anionic areas are repelled [12,13]. Thus, botulinum toxins are oriented towards the membrane in such a way as to make its cationic surface coincide with the electronegative electrostatic potential of raft gangliosides [14]. This initial orientation is decisive in promoting the fixation of the toxin to its membrane receptor, while preserving interactions with the gangliosides in the raft. It is the same phenomenon as electrostatic attraction, which controls the attachment of virus surface proteins to the host cell plasma membrane. Thus, very phylogenetically distant viruses, such as HIV-1 and SARS-CoV-2, are characterized by convergent evolution driven by the electrostatic surface potential [14]. In addition, the evolution dynamics of these viruses shows a progressive increase in the electrostatic surface potential, which can be quantified, in particular, in the successive variants of SARS-CoV-2 [15] and influenza viruses [16]. As a fundamental parameter of biology [4], the electrostatic surface potential controls many physiological and pathological mechanisms. However, its contribution to the control of synaptic transmission is notably underestimated. The calculation of the electrostatic potential of a lipid raft containing gangliosides (e.g., GM1 in Figure 2) shows a large electronegative zone that covers the entire surface of the raft.

If we consider that the gangliosides are raised relative to the average height of the other lipids in the plasma membrane [17], we see that the electronegative influence zone of the raft is exacerbated. A ligand that approaches the lipid bilayer of a membrane will, therefore, be very easily attracted (if it is cationic) or repelled (if it is anionic) by the raft.

Cationic neurotransmitters, such as serotonin or dopamine, are therefore strongly attracted to the post-synaptic membrane [18,19]. In the case of serotonin, whose solubility limit is lower than its concentration in synaptic vesicles, this electrostatic attraction contributes to the dissolution of serotonin aggregates present in the synaptic space [19]. For glutamate, the situation is reversed, since its very strong negative surface potential prevents any approach to the post-synaptic membrane. In fact, the electric field of the post-synaptic membrane generated by raft gangliosides is a particularly effective mechanism to prevent glutamate from reaching the post-synaptic neuron. It is likely that this mechanism was put in place during evolution to protect the post-synaptic neuron from glutamate excitotoxicity. This ganglioside-induced electronegative field is distinct from electric fields generated by synaptic currents, which have also been suggested to speed up the clearance of charged neurotransmitters from the synaptic cleft [20,21]. Such currents may transiently affect the relative charge of gangliosides, without reversing their surface potential, which remains electronegative in the presence of high Ca^2+^ concentrations [22]. Despite the fact that most electrogenic channels are located in lipid rafts [23], the impact of the electrostatic surface potential of ganglioside-rich microdomains is generally overlooked. To the best of our knowledge, this review is the first to consider this parameter in the global process of synaptic transmission. However, this concept was initially discussed in the fifth chapter (*A molecular view of the synapse*) of a book dedicated to brain lipids, published in 2015, by two of the authors of this paper (J.F. and N.Y.) [19].

## 4. Electrostatic Constraints Applied to Post-Synaptic Glutamate Receptors

**Figure 3:**
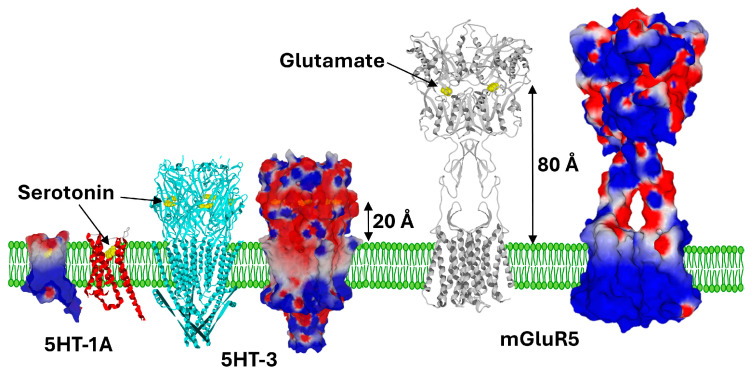
Membrane topology of serotonin and glutamate receptors. These structures were retrieved from PDB files 7e2y (5HT-1A) [26], 6dg7 (5HT-3) [27], and 6n51 (mGluR5) [28]. The neurotransmitters bound to the receptors are represented as yellow spheres. Each receptor is represented as ribbons to locate the transmembrane domains (red for 5HT-1A, cyan for 5HT-3, grey for mGluR5) and in surface rendition of the electrostatic potential (electronegative zones in red, electropositive in blue, neutral in white). The distance between the neurotransmitter binding site and the surface of the lipid bilayer is indicated beside black arrows.

We consider that the constitutive electric field of the post-synaptic neuron membrane is a key factor that determines the geometric adaptation of neurotransmitter receptors. For serotonin, which is attracted to the post-synaptic membrane, the extracellular part of the receptor occupies a small volume, since the neurotransmitter is delivered to the membrane by raft gangliosides [19]. The majority of serotonin receptors are G-protein-coupled receptors (GPCRs), with seven transmembrane domains [24]. These receptors are flush with the membrane and their serotonin binding site is located at the apolar third of the outer leaflet of the plasma membrane [25,26]. Under these conditions, the initial attraction of serotonin by raft gangliosides promotes the approach of the neurotransmitter to its receptor. However, we must also consider the case of the unique ionotropic receptor for serotonin, 5-HT3. The binding site of serotonin on this ion channel receptor is located in the extracellular part of the pentameric receptor [27]. It is interesting to note that this binding site is also relatively close to the lipid bilayer of the post-synaptic membrane. As shown in Figure 3, the 5-HT3 receptor is much smaller than ionotropic glutamate receptors. The distance between the highest part of the ganglioside and the binding site of serotonin is about 20 Å for the 5HT-3 receptor [27] compared to 80 Å for the binding site of glutamate on the metabotropic glutamate receptor, mGluR5 [28].

It is likely that this difference is due to the electric field generated by the gangliosides in the post-synaptic membrane, which attracts serotonin and repels glutamate. This repulsion phenomenon is especially spectacular for metabotropic glutamate receptors, which belong to the GPCRs family [28]. The neurotransmitter binding site of these receptors is unusual, since GPCRs generally have a small extracellular domain. There is, however, a notable exception to this rule, which concerns a particular category of GPCRs that possess a very large extracellular domain, reminiscent of the structure of carnivorous plants. By extension, these domains are called “Venus Flytraps” [29,30,31]. Those receptors are composed of a Venus Flytrap module, where the ligand binds, connected to a heptahelical domain responsible for G-protein coupling [32]. Figure 3 compares the respective sizes of glutamate and serotonin receptors. The logic that emerges from this comparative analysis is clear: serotonin binding sites are close to the post-synaptic membrane, while glutamate binding sites are much further away. We consider that the reason for this geometric adaptation is the electrostatic field produced by ganglioside-rich lipid rafts.

But it is not enough for a glutamate receptor to position the binding site of its neurotransmitter far from the post-synaptic membrane. It is necessary to attract glutamate towards its binding site in order to optimize the chances of stimulation and, therefore, to trigger synaptic transmission. Here again, it is an electrostatic mechanism that comes into play. In fact, glutamate is attracted towards its interaction site by an electrostatic funnel that directs the glutamate in the right direction. This electrostatic funnel has cationic walls that are well adapted to the transfer of glutamate from the synaptic space to the inside of the receptor. A similar mechanism has previously been demonstrated for the GABA receptor, a zwitterionic neurotransmitter also influenced by electric fields. In this case, the binding pathway of the neurotransmitter is driven by long-range electrostatic interactions, whereby the electrostatic field acts as a funnel that sweeps the GABA molecules towards the binding site, at which point more specific atomic interactions take over [33]. 

**Figure 4:**
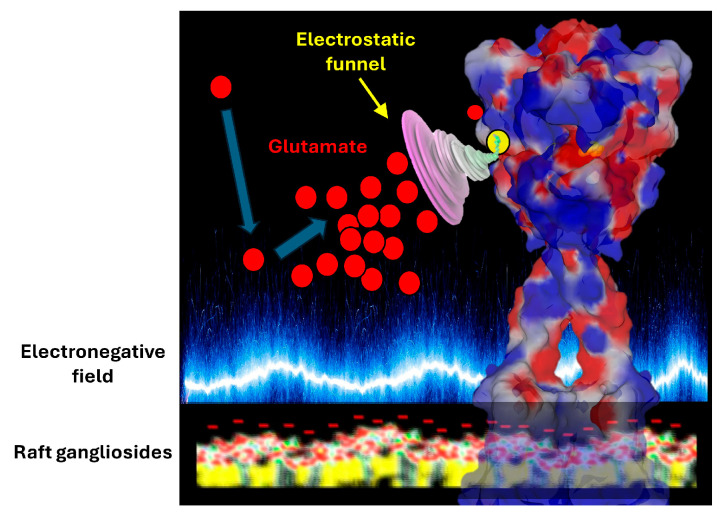
Glutamate molecules (represented as red disks) are repelled by the electrostatic surface of raft gangliosides. Then, they are attracted by the ligand binding domain of the receptor (blue arrows). This process may be facilitated by an electrostatic funnel, as suggested in regard to the GABA receptor [33]. The electrical surface potential (ΔV) of packed ganglioside monolayers (at a surface pressure π = 30 mN/m) varies from −17 mV for GM1 (one negative charge per molecule) to −39 mV for GT1b (three negative charges per molecule) [36].

Based on this analysis, we can conclude that glutamate, released at a high concentration by the synaptic vesicles [34,35], will first rebound on post-synaptic membrane gangliosides, which keep it at a safe distance from the post-synaptic neuron. It then joins the pool of glutamate present in the synaptic cleft, which is under the influence of the electrostatic funnel of the receptor (Figure 4). But only a few glutamate molecules released by the synaptic vesicles will stimulate the receptor and transmit the synaptic information. The vast majority are expelled from the synaptic space, which minimizes the potential toxicity of this excitatory neurotransmitter. This is where the notion of the tripartite synapse comes into play.

## 5. Electrostatic Control of Glutamate Flux in the Synaptic Cleft

**Figure 5:**
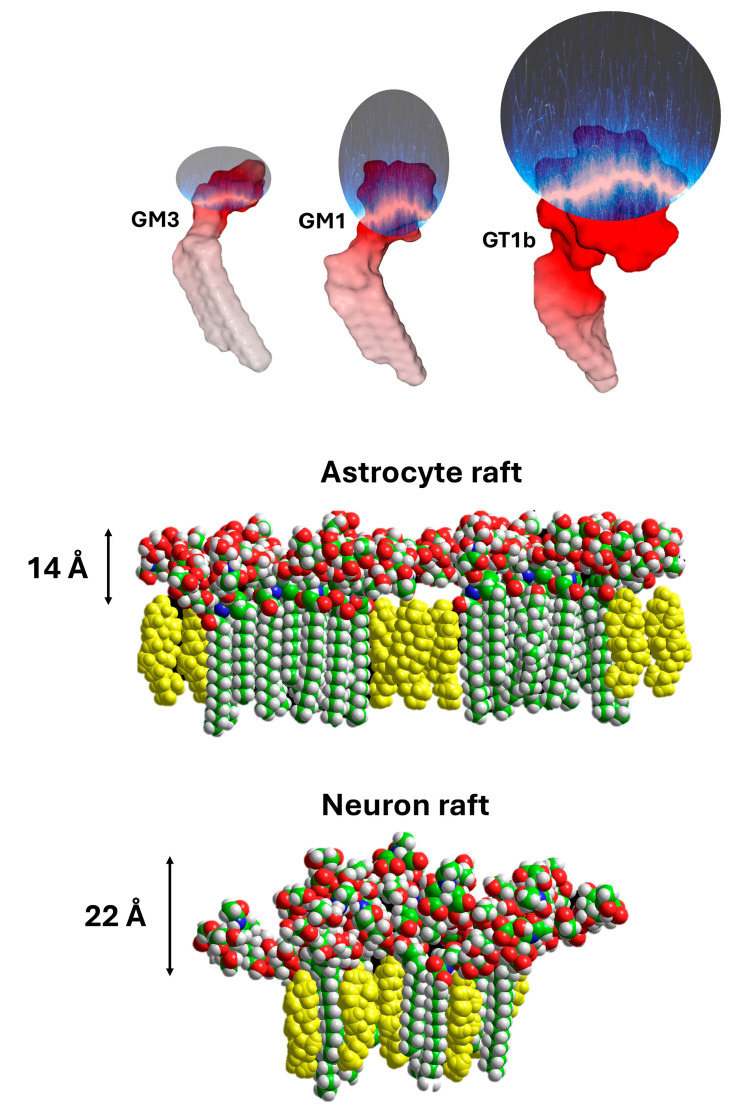
Specific content of gangliosides generates distinct lipid raft structures and electronegative fields in neurons and astrocytes. The strength of the electrostatic field of GM3, GM1, and GT1b gangliosides (**upper** panel) is based on quantitative measurements of the ganglioside monolayers [36,41]. The structure of astrocyte and neuron rafts are shown in the **central** and **lower** panels, respectively. Gangliosides are colored according to the atom names and cholesterol are in yellow. The mean height of the oligosaccharide parts of raft gangliosides is indicated on the left.

Even schematically, a synapse cannot be described by considering only the couple formed by the pre- and post-synaptic neurons. A third partner, fundamental for the balance of the system, intervenes, the astrocyte [37,38]. It is by combining the electric fields of these three cells that we understand how glutamate is first expelled from the synaptic space, then recycled. First, the membrane of the pre-synaptic neuron is itself enriched with gangliosides [39], which gives it a negative electrostatic surface potential. Glutamate cannot, therefore, return directly to the source of emission, the post-synaptic neuron. Subject to the dual influence of the electric fields of the pre- and post-synaptic neurons, it is naturally directed towards the least electronegative site present in the environment of the synapse. This site is none other than the plasma membrane of the astrocyte. Indeed, astrocytes also express gangliosides, but almost exclusively GM3 [40]. The chemical structure of GM3, which contains only two sugars and one sialic acid, is much smaller compared to those of the four main neuronal gangliosides, i.e., GM1, GD1a, GD1b, and GT1b [11]. Thus, one can perfectly differentiate a “neuronal raft” from an “astrocytic raft” (Figure 5). An estimation of the impact of the number of sialic acid units in gangliosides on the electrical potential has been given by Beitinger et al., who measured the surface potential (ΔV) of packed ganglioside monolayers: GMI = −17 mV, GT1b = −39 mV at a surface pressure π = 30 mN/m [36]. These negative values are in agreement with the electrostatic properties of ganglioside monolayers, as detailed by Maggio’s laboratory [41]. 

The consequence of this biochemical specificity is that the electrostatic field generated by GM3 on the surface of astrocytes is much weaker compared to that of neurons, which have larger gangliosides with one, two, or three sialic acids that are negatively charged at a physiological pH. The pathway of glutamate in the synaptic space is, thus, very clearly signposted and oriented towards the astrocyte. It is striking to note that this pathway from the pre-synaptic neuron to the astrocyte actually concerns the vast majority of glutamate molecules released into the synaptic space by the vesicles, since very few neurotransmitter molecules bind to their post-synaptic receptors. The loss is important (>90%) [20], but it is largely compensated by the glutamate recycling mechanism.

## 6. How Astrocytes Buffer Glutamate and Manage Its Recycling

Glutamate recycling begins with its transport into the astrocyte, which is mediated by a class of transporters called excitatory amino acid transporters (EAATs) [42]. These transporters operate according to a conformational mechanism called the “elevator” [43,44], which allows them to free themselves from potential electrostatic constraints of GM3 gangliosides present in the astrocyte membrane. One may wonder why glutamate, whose entry into the post-synaptic neuron is prohibited due to its toxicity, is favorably and massively received by the astrocyte. In fact, the astrocyte immediately detoxifies glutamate by transforming it into glutamine [45], which is zwitterionic and less toxic than anionic glutamate. In addition, the surface potential of glutamine is more balanced than that of glutamate, with a zero net charge. Incidentally, glutamine has no neurotransmitter activity. Glutamine is synthesized by an amidation reaction, catalyzed by glutamine synthetase, an enzyme that is restricted to glial cells. Glutamine is then safely released into the extracellular space, returned to neurons, and deaminated to produce glutamate, in a global glutamate–glutamine cycle [46]. This mechanism allows the pre-synaptic neuron to recover a large part of the glutamate released during exocytosis of synaptic vesicles.

## 7. Pathological Implications

The role of gangliosides in the brain and synaptic transmission goes well beyond the electrostatic properties of brain cell membranes described in this review. First, we must consider separately the functions performed by individual ganglioside molecules and those emanating from emergent properties linked to the concentration of these glycolipids in lipid raft microdomains [47]. But the situation is more complicated than it seems. For the individual properties of gangliosides, we need to consider the complex network of molecular interactions with neurotransmitter receptors and transporters. Indeed, the function of these membrane proteins is tightly controlled by gangliosides, which act by tuning their unbound conformation and facilitating the conformational changes induced by their ligands through chaperone activity [47]. The preferential localization of neurotransmitter receptors [48,49] and transporters [50] in lipid rafts facilitates these functional contacts, but at the same time complicates the analysis. Indeed, lipid rafts are divided into several areas containing several pools of gangliosides with distinct mobility properties. Typically, those gangliosides at the edge of a lipid raft have more conformational freedom than those located in the central zone of the raft [51]. At least two distinct sphingolipid binding domains (SBDs) have been characterized in membrane proteins, including neurotransmitter receptors and transporters [51,52]. Ganglioside binding to an SBD may stabilize the localization of these membrane proteins in lipid rafts, as shown for ionotropic glutamate receptors [52] and for the serotonin receptor, 5-HT1A [53]. It may also facilitate functional contacts with cholesterol molecules that are closely associated with gangliosides in the lipid raft and also control receptor function through direct binding [54,55,56]. 

Second, gangliosides may act collectively to generate an optimal physicochemical environment, materialized by favorable membrane fluidity and curvature [57,58], and also a functional electronegative field [36,59,60]. By combining all these parameters, several distinct pools of gangliosides can act as critical partners of synaptic transmission. Not surprisingly, knock-out (KO) mice lacking selected gangliosides revealed key neural functions for the complex gangliosides in the brain. In particular, these KO animals are highly susceptible to the severity and duration of kainate-induced seizures [61]. Kainate receptors are ionotropic glutamate receptors involved in the control of neuronal excitability [62]. Due to post-translational palmitoylation [63], kainate receptors are associated with lipid rafts, like the two other ionotropic glutamate receptors, AMPA and NMDA [49].

Interestingly, several studies have demonstrated a protective effect of extracellularly applied gangliosides against glutamate and kainate neurotoxicity [64,65,66,67]. Although several mechanisms may explain this neuroprotection [68], it is particularly significant that it does not require the presence of gangliosides in the incubation medium of cultured neurons [64]. Moreover, the effect is proportional to the amount of gangliosides accumulated in the neuronal membranes, and it is strictly dependent on the sialylation level: asialo-GM1 has no effect, whereas the maximal effect was obtained by GT1b (trisialylated), followed by GD1b (disialylated), and finally GM1 (monosialylated) [64]. In line, with these data, other studies have shown that neuroprotection is induced by the oligosaccharide part of gangliosides [69,70]. Certain aspects of neuroprotection induced by gangliosides are interesting to note. First, gangliosides are effective against both glutamate- and kainate-induced neurotoxicity, while they fail to block glutamate-gated cationic currents [64]. This indicates that gangliosides do not act by masking the transmitter recognition sites or by impairing the cationic channel activity of ionotropic glutamate receptors. Second, gangliosides do not seem to interfere with the transduction mechanism that couples the transmitter recognition site with phospholipase C [64]. Overall, gangliosides do not affect glutamate receptor operation, but inhibit the consequences of uncontrolled and persistent glutamate receptor activation. One possibility is that the insertion of ganglioside into the plasma membranes of cultured neural cells incubated with gangliosides may induce a stronger electronegative field that would provide a protection shield against glutamate neurotoxicity. An argument in favor of this concept is the greater effectiveness of ganglioside GT1b compared to GD1b and GM1 in treating glutamate neurotoxicity, which corresponds to the strength of their respective electrostatic surface potential [36]. Clearly, this possibility warrants further consideration and specific experimental studies.

Among the neurological disorders involving gangliosides, we can cite Alzheimer’s disease, Parkinson’s disease, Rett syndrome, Huntington’s disease, amyotrophic lateral sclerosis (ALS), and hereditary spastic paraplegia (HSP) [51,71,72,73,74]. In the first two cases, gangliosides play an active role in the attachment of the amyloid proteins responsible for these diseases to the lipid rafts of brain cells [75,76]. This facilitates the insertion of these proteins into the outer layer of the plasma membrane, and then the formation of neurotoxic oligomers controlled by cholesterol. Blocking the binding of these proteins to gangliosides is, therefore, a possible therapeutic approach [77]. This strategy takes into account the fact that the pool of gangliosides accessible to amyloid proteins is distinct from that associated with neurotransmitter receptors and transporters, through specific molecular interactions between gangliosides and SBDs [51]. Interestingly, GM1 and its oligosaccharide have been shown to reduce α-synuclein aggregation and to protect dopaminergic neurons from α-synuclein toxicity in several models of Parkinson’s disease [78,79]. As previously discussed, extracellular GM1 (or GM1 oligosaccharide) may bind to α-synuclein and inhibit both its prion-like propagation and the formation of Ca^2+^ permeable oligomeric pores in brain cells [76]. Whatever the mechanism of action, GM1 might thus be considered as a disease-modifying therapy for Parkinson’s disease [80]. In the case of Rett syndrome, the link with gangliosides is proven, but still remains mysterious at present [81]. On the one hand, we have an excess of glutamate [82] and, on the other hand, we have a deficit in the expression of brain gangliosides [83]. The ganglioside pattern of patients with Rett syndrome revealed an increase in astroglial cell-associated gangliosides and reduced proportions of gangliosides GD1a and GTlb [83]. These modifications are expected to affect the electrostatic properties of the tripartite synapse and, thus, the synaptic half-life and clearance of glutamate. This would logically increase the amounts of glutamate in the brain and, secondarily, in the cerebrospinal fluid (CSF). Accordingly, precise measurements revealed a mean CSF glutamate concentration of 355.2 nmol/L (± 109.2 nmol/L) in patients with Rett syndrome, compared with 203.9 nmol/L (±55.5 nmol/L) in the controls [82]. Such an increase in CSF glutamate indicates the enhanced release of glutamate from pre-synaptic terminals [84] or a defect in glutamate clearance from the tripartite synapse, according to the concept developed in the present review. In both cases (which are not mutually exclusive), the excess of glutamate in the synaptic cleft provides a plausible explanation for the seizures, EEG abnormalities, and respiratory irregularities in Rett syndrome [84]. Any therapeutic strategy aimed at buffering glutamate in the synapse may thus be relevant to target the symptoms of Rett syndrome. In this respect, gangliosides or oligosaccharides derived from gangliosides [74] are obviously interesting options. Finally, gangliosides are also involved in amyotrophic lateral sclerosis (ALS) [70,85,86] and hereditary spastic paraplegia (HSP) [87,88] and may, thus, represent appropriate therapeutic targets for those diseases.

## 8. Consideration of the Electrostatic Parameter of Lipid Rafts: Overview of the Literature

One may wonder why this field of research, although well documented since the end of the 1970s, has not progressed more rapidly. Several explanations can be put forward. First of all, the recognition of lipid rafts as functional signaling platforms only occurred in 1997, after the seminal article by Simons and Ikonen [9]. It was only from this date that gangliosides, which are major raft components, could be considered by cell biologists not individually but collectively, emphasizing their emergent properties. The pioneering studies on ganglioside monolayers at the water–air interface [36,41,59] were then highlighted and reinterpreted in the context of the newly emerging raft concept, bringing together biophysics and cell biology fields [89,90]. Yet in the early ages of the raft theory, most efforts were devoted to demonstrating the actual existence of lipid rafts in vivo [91]. In parallel, many laboratories around the world have contributed to provide an inventory of proteins associated with rafts [92]. We thus quickly found ourselves in a simplified binary model, minimizing the emergent properties of raft lipids. Another problem is that gangliosides combine two experimental difficulties, due to their dual lipidic and glycosidic nature. No ganglioside is encoded by a single gene, which differentiates them from proteins, which remain the biomolecules preferentially studied by biologists. Finally, the electrostatic surface potential is a neglected parameter of biology absent from most textbooks [4], despite the fact that bioelectric fields play a primordial role in many biological mechanisms [93] and, especially, in the brain [94]. Indeed, studies on the surface behavior of raft lipids in biomembranes, which now span over four decades, allowed the electrostatic properties of gangliosides to be described and for their area of influence, as well as their biological impact, to be specified [41,59,60,95,96,97,98,99].

## 9. Conclusions and Perspectives

Overall, one can see that the chemical structure of neurotransmitters and their electrostatic surface potential is closely associated with receptor-binding mechanisms. The net electric charge of the neurotransmitter determines whether its binding site is located beyond the influence of the negative electrostatic field generated by lipid raft gangliosides (as is the case for glutamate), or close to the membrane or even inside the membrane (in the case of serotonin). In this respect, the selection of Venus Flytrap extracellular binding domains for metabotropic glutamate receptors is a logical solution to counteract the strong electronegative field of post-synaptic lipid rafts. Brain gangliosides act as auxiliary factors helping the neurotransmitter to find its way to the post-synaptic membrane, thereby speeding up the whole process of synaptic transmission. The differential expression of gangliosides in the three cellular partners of the tripartite synapse drives the clearance and recycling of excess glutamate, which is irreversibly directed toward the astrocyte and then recycled through a glutamate–glutamine cycle. The consideration of the role of electrostatics in synaptic mechanisms may inspire innovative therapeutic solutions for neurological diseases that are associated with an impairment of brain ganglioside functions. 

## Data Availability

No data were generated for this review.
